# Analysis of nocturnal, hypoxia-induced miRNAs in sleep apnea patients

**DOI:** 10.1371/journal.pone.0263747

**Published:** 2022-03-04

**Authors:** Philip Roger Goody, Lisa Nachtsheim, Mohammed Rabiul Hosen, Miriam von Krosigk, Dominik Christmann, Jens Peter Klussmann, Andreas Zietzer, Nils Breitrück, Felix Jansen, Stefanie Jansen

**Affiliations:** 1 Department of Medicine II, Heart Center Bonn, University Hospital Bonn, Germany; 2 Department of Otorhinolaryngology, Head and Neck Surgery, University of Cologne, Cologne, Germany; Brigham and Women’s Hospital and Harvard Medical School, UNITED STATES

## Abstract

**Introduction:**

Obstructive sleep apnea syndrome (OSAS) is associated with an increased cardiovascular risk. The underlying mechanisms are largely unclear. MicroRNAs (miRNAs) are RNAs circulating in the blood that can be released into the bloodstream during hypoxia. In the present study, we investigate if OSAS-induced hypoxia results in a release of miRNAs that may mediate OSAS-associated cardiovascular damage.

**Methods:**

Blood was sampled from 23 OSAS patients before and after a polygraphically monitored night. Total circulating RNA was isolated from the plasma and quantified using real-time qPCR. Using a Taqman miRNA array, the levels of 384 different miRNAs were compared between evening and morning after polysomnography. The most highly upregulated miRNA (miRNA-505) and four additionally upregulated miRNAs (miRNA-127, miRNA-133a, miRNA-145, and miRNA-181a) were then quantified in a bigger patient cohort individually.

**Results:**

Apnea/Hypopnea-Index (AHI) was evaluated and averaged at 26 per hour on nocturnal polygraphy. In an initial miRNA array, a total of 4 miRNAs were significantly regulated. A significant increase of miRNA-145 was observed in the larger patient cohort. No significant changes in concentration were detected for miRNA-127, miRNA-133a, miRNA-181a, and miRNA-505 in this larger cohort.

**Conclusion:**

OSAS results in the nocturnal release of miRNAs into the bloodstream. Our collected data may indicate a hypoxia-induced release of miRNAs into the bloodstream of OSAS-patients. *In vitro* experiments are needed to confirm the secretion of these miRNAs under hypoxia and evaluate the effect on the cardio vasculature.

## 1 Introduction

Obstructive sleep apnea syndrome (OSAS) is a sleep-related breathing disorder that occurs in large proportions of the population. It is by far the most common sleep disorder with a rising prevalence, accounting for 85% of all cases of sleep apnea [1]. OSA is caused by a blockage of the upper airway during existing respiratory effort. In OSA patients, a collapse of the pharyngeal muscles leads to phases of reduced oxygen supply followed by brief arousals. Typical symptoms to be expected in OSA patients include heavy, irregular snoring and pronounced daytime sleepiness, breathing cessation during sleep, lethargy and reduced work performance [2]. Risk factors for the development of OSA include obesity/high BMI (body mass index), alcohol consumption, craniofacial anatomy, age, gender, smoking and family history [1, 3]. In the definition of OSA, a distinction is made between apnea and hypopnea phases. In apnea phases, there is a reduction in respiratory flow of at least 90% over a period of at least 10 seconds. Hypopnea phases are defined as a reduction in respiratory flow of more than 30% for more than 10 seconds, combined with an oxygen desaturation of at least 4% or an arousal [1, 4]. The apnea-hypopnea index (AHI), which measures apnea and hypopnea phases per hour of sleep, is used to classify the severity of OSA. The diagnosis of OSA can be made with an AHI>5 in combination with typical symptoms. An AHI of 5–15 is considered to be mild. Values between 15–30 indicate a moderate OSA, while an AHI of more than 30 can be diagnosed as severe OSA [3]. Treatment options for patients with OSA include the use of continuous positive airway pressure (CPAP), variable/bilevel positive airway pressure (BiPAP), dental appliances and surgeries such as uvulopalatopharyngoplasty (UPPP) or maxillomandibular advancement (MMA) [1].

The association of OSA with hypertension, heart failure, coronary artery disease and atrial fibrillation has been repeatedly reported. In patients with severe OSA (AHI>65) and severe oxygen desaturations (SaO2 <65%), the risk of cardiac arrythmias increases [5, 6]. Furthermore, the increased cardiovascular mortality of any cause, an increase of the risk of stroke, diabetes and hypertension have been associated with untreated OSA [7, 8]. Additionally, it has been shown that 50% of patients with OSA have a history of hypertension, and between 30–40% of patients with hypertension had OSA when tested for it [5]. The exact underlying pathomechanisms regarding increased cardiovascular risk are still largely unclear. It has been suspected that intermittent hypoxemia and re-oxygenation, arousals and the resulting changes in intrathoracic pressure contribute to the pathomechanisms triggered by OSA [5].

MicroRNA (miRNA) are short, non-coding RNAs with a length of 19 to 23 nucleotides which are known for key gene regulatory activities in numerous contexts [9]. MiRNAs are not converted into protein products, but rather regulate the conversion of genes to protein products and have thus been suspected of being involved in the pathogenesis of diseases such as primary hypertension. In many types of cancer, hypoxia is a hallmark of the tumor microenvironment and can trigger the release of miRNAs into the extracellular matrix and the bloodstream [10]. Patients with OSA have been shown to display a dysregulated miRNA profile, when compared to non-OSA controls [11], however an analysis of microRNA profiles in OSA patients before and after a polographically-monitored night, has not yet been undertaken. It is likely that intermittent nocturnal hypoxia, as displayed by patients with OSA, can lead to a differential secretion of hypoxia-induced miRNAs.

## 2. Material and methods

### 2.1 Study population

23 patients with suspected OSA were included in this study. Blood was drawn from all patients before and after a polygraphically monitored night. One patient did not meet the OSA criteria and was excluded from further analysis, thus 22 patients were further investigated. Written informed consent was obtained from all patients. The study protocol was approved by the ethics committee of the University Hospital Cologne (No. 19–425).

### 2.2. Sample acquisition and preparation

Citrate blood was drawn on the evening before and the morning after a polygraphically monitored night from a peripheral vein. The sample was transported to the laboratory on ice and centrifuged 2 times at 2500 g (4°C, 15 min) to remove cells and debris and once more at 3000 g (4°C, 15 min) to generate platelet-free plasma. Citrate plasma samples where then frozen and stored at—80°C until further analysis.

### 2.3. Total blood RNA isolation and polymerase chain reaction

Total blood RNA was isolated with a TRIzol (#15596026 ThermoFisher Scientific) and phenol-based protocol. After purification RNA was diluted in DNAse/RNAse free water and RNA concentration was measured via NanoDrop (ThermoFisher Scientific). For reverse transcription we utilyzed TaqMan microRNA Reverse Transcription Kit (#4366596, ThermoFisher Scientific) and microRNA specific primers. Quantitative polymerase chain reaction was carried out on 7900HT thermo cycler (Applied Biosystems). CDNA was diluted to 1ng/μl and 9μl were pipetted per well. 1μl primer was mixed with another 10μl of Universal MasterMix II (#44-400-49 ThermoFisher Scientific). CT values above 40 cycles were defined as undetectable. Data was analysed using the ddCT method.

### 2.4 miRNA analysis

5 patients were selected randomly for initial array experiments. RNA was converted to cDNA by priming with a mixture of looped primers (MegaPlex PreAmp Primers, Human Pool; MegaPlex RT primers, Human Pool; Applied Biosystems, Foster City, CA, USA). TaqMan Array miRNA Cards (#4444913, Applied Biosystems) were used containing a total of 384 unique assays specific to human miRNAs under standard qRT-PCR conditions. qRT-PCR was carried out on an Applied Biosystems 7900HT thermocycler using the manufacturer’s recommended program. Analysis of data was performed using Expression Suite V1.1 program (Applied Biosystems). Cycle of threshold (CT) values above 40 were defined as undetectable. U6snRNA was chosen as housekeeping gene, due to stable expression across all samples. CT values were exported to Microsoft excel (Microsoft Corporation, Albuquerque, NM, USA) and CT and ddCT levels were calculated. Evaluation of results was performed with GraphPad Prism 7 (GraphPad Software Inc., San Diego, CA, USA). Statistical analysis was performed using multiple T-Testing with Bonferroni-Dunn method for multiple comparisons and detection of false positives.

### 2.5. Statistical analysis

For statistical analysis, GraphPad Prism was applied. Mann-Whitney significance testing was performed for patient samples. Error bars in graphs indicate standard error of the mean (SEM).

### 2.6 Ethics statement

Written informed consent was obtained from all patients. The study protocol was approved by the ethics committee of the University Hospital Cologne (Nr. 19–425).

## 3. Results

### 3.1 Patient cohort and baseline characteristics

**Fig 1:**
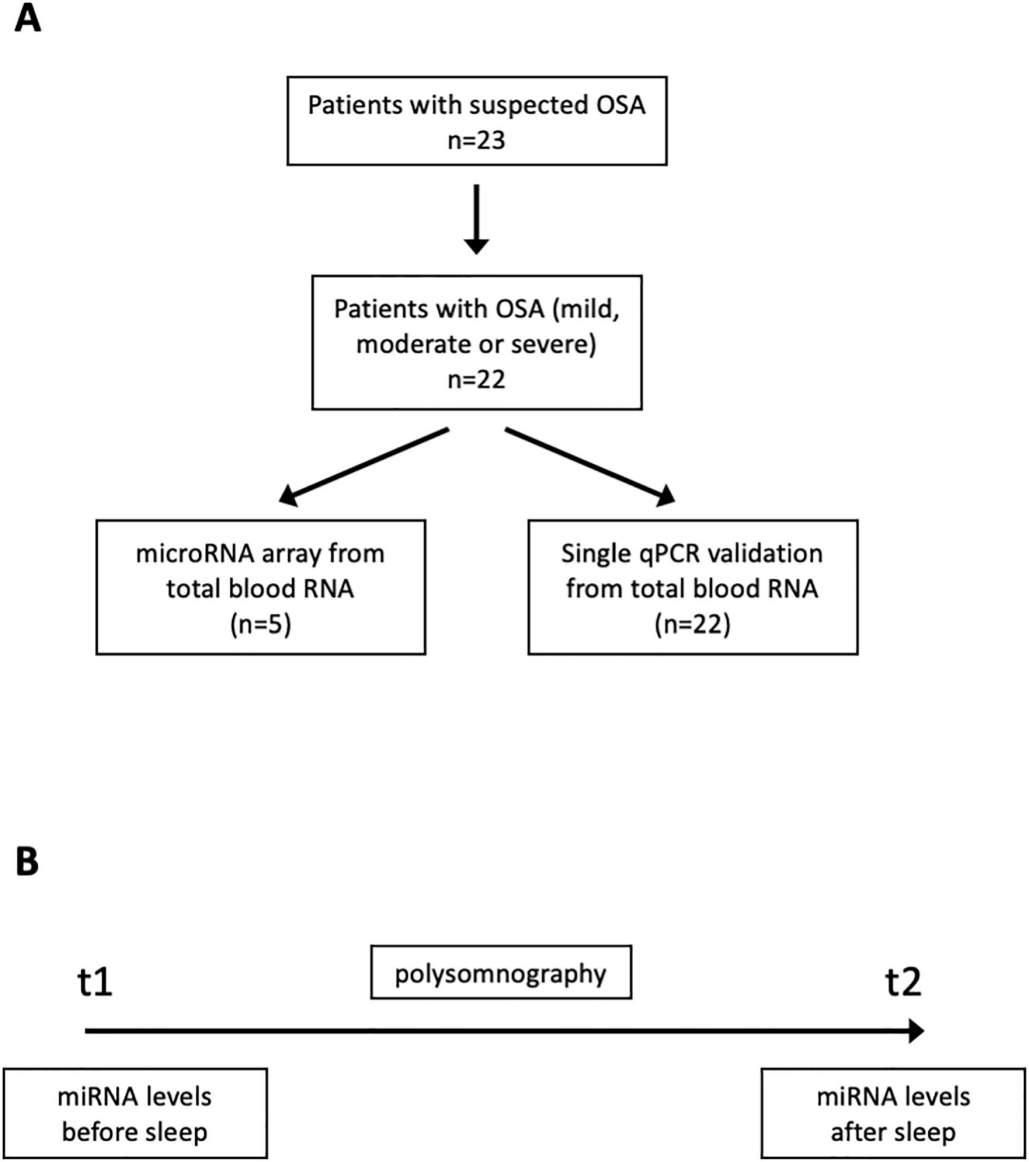
A Study design, B timeframe of analysis: First peripheral blood sample at t1 (before night), second peripheral blood sample at t2 (after night).

One patient did not meet the study criteria and was excluded from the analysis. 19 male and 3 female patients were included in this study (Table 1, Fig 1). The mean age was 44 and the mean AHI 28/h. The mean ESS score was 9,9 with an IQR of 6,5. In the polygraphy, 4 patients (18,2%) were diagnosed with mild OSA (AHI 5-15/h). 8 patients (36,4%) were diagnosed with moderate OSA (AHI 15-30/h) and 10 patients (45,5%) with severe OSA (>30). The mean oxygen desaturation index (ODI) was 21,7/h with a minimum oxygen saturation (O2 sat nadir) of 79,1%. Most patients in this study were overweight, 7 patients had a BMI >30 kg/m^2^. Only 4 patients had a BMI below 25 kg/m2 and the mean BMI of all patients was 28,7 kg/m^2^. 6 patients had pre-existing cardiovascular risk factors: 5 patients had been diagnosed with arterial hypertension, 1 patient had a coronary artery disease and 1 patient had peripheral arterial disease. 4 patients that were included in this study were smokers.

**Table 1:** Baseline characteristics.

Demographic summary			
	Number (%)	Median Age (y)	BMI
Total	22	44	28,7 kg/m^2^
Female	3 (14%)	30,2	30,1 kg/m^2^
Male	19 (86%)	44,1	28,4 kg/m^2^
**Polysomnography findings**			
mean ESS	9,9	IQR ESS	6,5
mean AHI (/hr)	28,3	IQR AHI	21,55
mean ODI (/hr)	21,7	IQR ODI	19,125
mean O2 Sat Nadir (%)	79,1	IQR O2 Sat nadir	9,25
**OSA Severety**			
	Mild	Moderate	Severe
	4 (18%)	8 (36,4%)	10 (45,6%)
**Cardiovascular Riskfactors (n)**			
art. Hypertension	5		
Hyperlipoproteinemia	0		
Diabetes	0		
CAD	1		
PAD	1		
Smoking	4 (5–20 py)		
**Laboratory parameters**			
	mean		
Kreatinin (mg/dl)	0,92		
GFR (mL/min)	96,7		
Leucocytes (x1E9/l)	7,7		
CRP (mg/l)	6,7		
**Medications on admission (n)**			
ACE inhibitor	4		
Angiotensine receptor blockers	2		
Beta blockers	3		
Calcium channel blockers	2		
Diuretics	2		
Statins	3		
Nitrates	1		

### 3.2 MiRNAs are dysregulated in patients with sleep apnea

**Fig 2:**
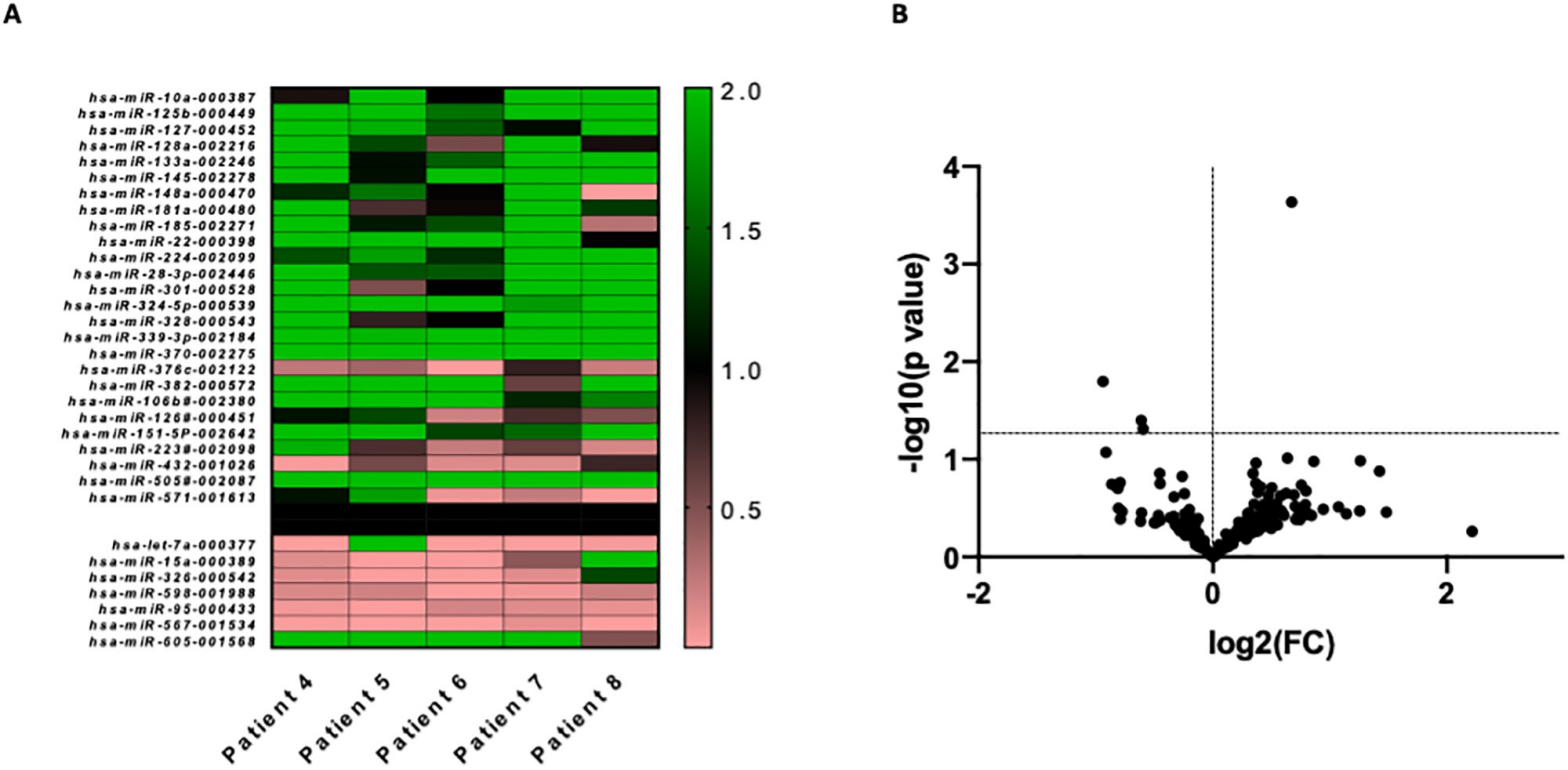
Micro RNA array from total blood RNA, n = 5. A heat map (shortened for better overview), B. Volcano plot.

Peripheral patient blood samples were obtained before and after sleep and polysomnographic analysis to evaluate hypoxia-induced miRNAs. Five patients were included for initial miRNA array analysis. Patients displayed a significant increase of one (miRNA-505) miRNA and a significant decrease of 3 circulating miRNAs (miRNA-598, miRNA-326, miRNA-432) (Fig 2). The significantly upregulated miRNA (miRNA-505) as well as four further, upregulated miRNAs (miRNA-127, miRNA-133, miRNA-145 and miRNA-181) were selected for validation in a larger cohort of patients.

### 3.3 Validation of miRNA candidates

**Fig 3:**
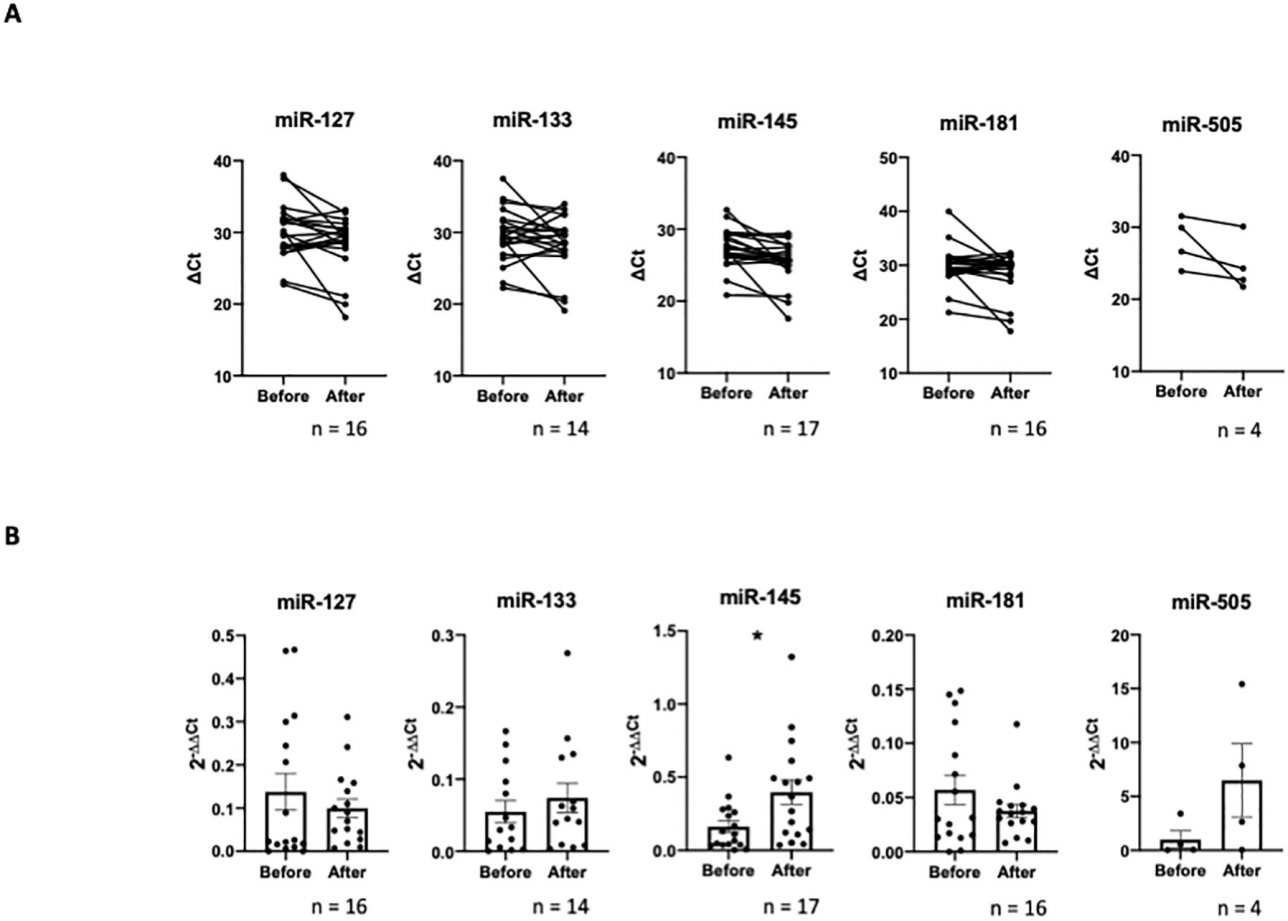
QPCR validation of identified miRNA targets. **A Individual miRNA changes displayed as CT-Values before and after the polygraphically monitored night. B Relative miRNA expression**. Different patient numbers due to outlier deletion, undetectable targets or low RNA input amount. All values displayed ± SEM, Outlier test Prism8; Mann-Whitney significance test, * P<0.*05*.

To examine the levels of the selected miRNA candidates, single reverse-transcription quantitative polymerase chain reaction (RT-qPCR) analyses were performed in a larger patient cohort (n = 22) (Fig 3B and 3C). In this cohort, the levels of circulating miRNA-145 were significantly upregulated. There was no significant dysregulation of the other four candidates, including miRNA-505.

## 4 Discussion

Obstructive sleep apnea is clearly associated with cardiovascular disease and mortality, but the underlying pathomechanisms are yet poorly understood [12]. Hypoxic conditions are able to trigger the release of miRNAs from mother cells into the extracellular matrix or the blood stream [10]. MiRNAs are non-coding RNAs, which can alter the gene expression of the parent cell by inhibiting protein transcription via binding to target mRNA, leading to its degradation via the RNA-induced Silencing Complex (RISC) [13]. MiRNAs can be secreted in the blood stream (either via extracellular vesicles or bound to RNA-binding proteins like Ago-2) and then be taken up by target cells, where they can affect genotype as well as phenotype by altering the target cells protein translation [13, 14]. MiRNAs can therefore function as paracrine/endocrine signaling molecules [15]. The involvement of miRNAs in the pathogenesis of many cardiovascular diseases has been proven in the last years [16]. Especially endothelial and vascular smooth muscle cell functions can be altered through dysregulated miRNA expression [17]. Since OSA has been shown to be an independent risk factor for multiple cardiovascular disorders, and a hypoxic desaturation is a major attribute of OSA, we sought to analyze the circulating miRNA levels in patients with OSA before and after a polygraphically monitored night to determine the changes in miRNA profiles after intermittent nocturnal hypoxia. Studies, investigating miRNA profiles in patients with OSAS as a potential biomarker of disease, have been undertaken. Santamaria-Martos and colleagues identified 6 differentially expressed miRNAs in patients with OSAS, when compared to patients without OSAS. Furthermore, normalization of these miRNAs after 6 months of continuous positive airway pressure (CPAP) treatment could be observed [11]. Freitas *et al*. sought to link differentially expressed miRNAs in patients with OSAS to heart failure, myocardial ischemia, and cancer proliferation. In this study, severe OSA was independently associated with the levels of circulating miRNAs known to be involved in cancer cell proliferation, heart failure, and myocardial ischemia/reperfusion (miR-1254, miR-320e; miR-1254; and miR-320e, respectively) [18]. In a study analyzing expression profiles of three cardiac-specific biomarkers (miR-1-3p, miR133-3p, miR-499) only miR-499 showed the potential of being used as a biomarker for disease. Limitations of this study are the lack of unbiased screening methods (e.g. next generation sequencing/miRNA array) for the identification of potentially useful biomarkers [19]. Howerver, to our knowledge, no study has investigated the nocturnal changes of circulating miRNA profiles in patient before and after a polysomnographically monitored night. Of the 23 included patients included in this study, 22 patients displayed an AHI allowing the diagnosis of OSAS (4 mild, 8 moderate, and 10 severe). Initial miRNA arrays from 5 patients blood samples revealed a differential regulation of circulating miRNAs before and after the polygraphically monitored night. MiRNA-505 was significantly upregulated and miRNA-598, miRNA-326, miRNA-432 were significantly downregulated (Fig 2). To validate our findings, we chose the only significantly upregulated miRNA (miRNA-505) as well as four other promising candidates (miRNA-127, miRNA-133a, miRNA-145, miRNA-181b) that were upregulated in the initial array, but failed to achieve statistical significance herein, for further analysis in the whole OSA cohort (n = 22). MiRNA-127 has been shown to protect proximal tubule cells against ischemia/reperfusion injury [20]. MiRNA-133a has been identified to mediate hypoxia-induced apoptosis in cardiomyocytes [21], while hypoxia induced miRNA-181b was shown to regulate angiogenesis of retinoblastoma cells [22]. In human umbilical vein endothelial cells (HUVECs), hypoxia-induced changes in miRNAs levels also included miRNA-505 [23]. Especially the downregulation of miRNA-145 has been found to be an independent factor regarding the pathogenesis of primary hypertension [24]. In patients with pulmonary hypertension, circulating miRNA-145 levels are increased, and mice that have been exposed to hypoxia also display an increase in circulating miRNA-145 [25]. In our validation cohort, we identified miRNA-145 as the only significantly upregulated miRNA. MiRNA-145 belongs to the miRNA-143/145 cluster and is a well-studied miRNA, which can act on multiple cells and exert beneficial or detrimental effects. Dysregulation of the cluster, or one of its members, occurs during essential hypertension, atherosclerosis, coronary artery disease (CAD) and pulmonary arterial hypertension [25–28]. Patients with stable CAD show lower levels of circulating miRNA-145 than healthy controls, but patients with unstable angina display elevated levels and miRNA-145 levels correlate with infarct size during myocardial infarction [29–31].

In regards to hypoxia, miRNA-145 has been shown to function as cardio-protective in myocardial ischemic injury by ameliorating inflammation and apoptosis by negatively regulating CD40 [32]. MiRNA-145 was also shown to be upregulated in cardiomyocytes after hypoxic treatment and seems to protect cardiomyocytes from apoptosis. On the other hand, when exposing a bladder cancer cell line (RT4 cells) to hypoxic conditions, the most upregulated miRNA was miRNA-145. This miRNA is not only a direct target of hypoxia inducible factor 1α (HIF-1α), but also harbors 2 hypoxia response elements upstream of the miRNA transcript [33]. Overexpression of miRNA-145 in this cell line led to an increase in apoptosis in normoxic conditions and inhibition of miRNA-145 decreased apoptosis under hypoxic conditions, demonstrating a role for miRNA-145 in hypoxia-induced apoptosis [33].

Mechanistically, it is currently unclear, if miRNA-145 exerts a beneficial or detrimental effect on the cardio vasculature in patients with OSAS. Furthermore, due to the small sample size of the overall study cohort, our findings will have to be validated and analyzed in a larger patient cohort. In-depth mechanistic studies are necessary to identify a possible role of miRNA-145 during the pathogenesis of OSAS-associated cardiovascular disease.

## 5 Conclusion

Circulating miRNAs are dysregulated after nocturnal intermittent hypoxic events in patients with OSAS. We identified miRNA-145 to be significantly upregulated in patients with OSAS. Further mechanistic studies have to be performed to elucidate a possible function of miRNA-145 in the context of OSAS-associated cardiovascular disease.

## Supporting information

S1 Data (XLSX)

S2 Data (XLSX)
